# Estimation of the real burden of invasive meningococcal disease in Argentina

**DOI:** 10.1017/S0950268819002024

**Published:** 2019-11-29

**Authors:** J. A. Gómez, P. Wetzler Malbrán, G. Vidal, M. Seoane, N. D. Giglio

**Affiliations:** 1 GSK, Buenos Aires, Argentina; 2 Ricardo Gutierrez Buenos Aires Children's Hospital, Buenos Aires, Argentina

**Keywords:** Argentina, bacterial meningitis, invasive meningococcal disease, *Neisseria meningitidis*, surveillance system, underreporting

## Abstract

Among the different existing types of bacterial meningitis, the one caused by *Neisseria meningitidis* is the main presentation of invasive meningococcal disease (IMD). IMD is a significant public health concern and has a reported incidence rate in Argentina of 0.44 cases per 100 000 inhabitants in 2015. However, the actual incidence is thought to be higher as passive surveillance systems neither report nor identify 100% of all cases. The aim of this study is to develop an estimation of the burden of IMD in Argentina closer to reality by adjusting/correcting several limitations observed in the surveillance data available. A retrospective observational study has been performed using four Argentinean national databases recording the number of IMD cases and deaths, serogroups of *N. meningitidis* and ages, between 2007 to 2016. The reported data were adjusted to account for underreporting and to also integrate the cases missed due to well-known limitations associated with the diagnosis of *N. meningitidis* detection methods. Data were further analysed by serogroups of *N. meningitidis* and by age groups. After these adjustments, the potential numbers of IMD cases and IMD-related deaths are estimated to be 3.1 and 1.9 higher than reported, respectively. The study corrects the previous underestimation of the disease burden and provides expectedly more robust estimates aligned with international evidence and highlights the importance of active surveillance, with high-quality methods, for a better definition of preventive strategies against IMD in Argentina.

## Introduction

Among the different existing types of bacterial meningitis, the one caused by *Neisseria meningitidis* is a serious disease associated with high mortality, survivors frequently keep severe and irreversible sequelae and it is one of the most frequent presentations of invasive meningococcal disease (IMD) [1]. IMD is a significant public health concern with incidences largely varying geographically, ranging from 0.5 to 6.0 cases per 100 000 inhabitants in developed countries to up to 1000 cases per 100 000 inhabitants during outbreaks in sub-Saharan Africa [2]. Incidence rates (IRs) of IMD also vary by age groups, with the highest rates of associated meningitis and sepsis in children aged <1 year old (y) [2, 3]. In Latin American countries, IMD IRs vary largely from one country to another, in a range of <0.1 to 2 cases per 100 000 inhabitants [4]. In Argentina, the IR of IMD was 0.44 cases per 100 000 inhabitants in 2015, based on a passive surveillance system [5].

Among the 12 classified serogroups of *N. meningitidis*, six (A, B, C, W, X and Y) account for most of the IMD worldwide but their distribution varies both in time and space [1, 4]. In Latin America, serogroups B and C predominated largely during the last two decades but with great variation between countries [4]. For instance, in Brazil, the predominant serogroup since 2002 is serogroup C [4]. Serogroup distribution is also dynamic, varying across time and during outbreaks. In Argentina, where serogroup B used to be predominant, changes have been observed with an outbreak associated with serogroup C during the 1990s and a consistent emergence of serogroup W in all the countries since 2006 [5–7].

Although the reporting of IMD is mandatory throughout Latin America, the surveillance is mainly passive and only a few countries have set up active surveillance systems [8]. Besides, access to facilities remains difficult in several countries of Latin America, affecting the quality of the detection methods. Furthermore, different meningococcal case definitions are used, and the diagnostic methods differ [4]. In 2011, the Global Meningococcal Initiative (GMI), a group of expert scientists and clinicians, held a meeting with Latin American representatives to discuss the burden of IMD and agreed that the true burden of IMD was largely underreported [4]. The GMI issued a few recommendations to improve the surveillance of IMD: harmonisation of data via quality controls, use of standardised diagnostic protocols, collaboration between high- and low-resource regions to improve laboratory capacity, implementation of active surveillance and use of polymerase chain reaction (PCR) in diagnosis [4].

On the other hand, the standard method previously used to diagnose IMD infections was the bacterial culture. However, early use of antibiotics and improper transportation of samples could lead to over 50% of potential IMD cases missed. Improving the quality of bacterial isolation and using the most sensitive identification methods are key factors to identify the real burden of IMD [9]. The GMI encourages the use of the PCR, and specifically real-time PCR (qPCR) in addition to bacterial culture diagnosis, as it is a faster and more sensitive method [4, 10]. Nevertheless, in many regions, many hospitals cannot afford PCR, explaining one cause of IMD underreporting [10]. The PCR is a molecular method that has been successfully used for bacterial meningitis diagnosis in developing countries. At Instituto Adolfo Lutz in São Paulo State, Brazil, the use of qPCR in the Public Health surveillance routine showed a significant increase of 85% in the detection of *N. meningitidis*, and consequent reduction of non-determinate meningitis cases [11].

Although the problem of underreporting is well known and some steps have been taken for improvement and standardisation of surveillance procedures to increase reporting, there is still considerable variation between reported IMD IRs throughout the Latin American region [4, 12]. In Argentina, over the last two decades, the IRs of reported IMD in several studies varied widely, between 2.60 cases (in 1993) to 0.30 cases per 100 000 inhabitants in 2010 [7, 8, 13–21]. The IR in infants <1 y is often considerably higher than the incidence for all-age groups at the national level. In 2015, the Directorate of Immunopreventable Diseases of the Ministry of Health reported an IR of 14.60 per 100 000 inhabitants in children <1 y for the period 2012–2014 [21]. In January 2017, the National Immunisation Program of Argentina introduced the meningococcal ACWY vaccination for infants (MenACWY-CRM) and the guideline for meningococcal vaccination developed by the Ministry of Health mentioned the occurrence of 175 IMD cases in 2015 with an incidence of 0.44 cases per 100 000 inhabitants and 13.2 cases per 100 000 infants <1 y [5, 13].

The substantial variation of reported IMD IRs between age groups and regions in Argentina makes the actual burden of the disease difficult to estimate. Clearly, there is a substantial underestimation of the currently reported burden of IMD in Argentina. It is important to improve estimations of IMD incidence to allow evidence-based decision making during the development and implementation of vaccination programs [20]. This study aims to give a robust estimation of the real burden of IMD in Argentina by correcting the effects of partial coverage of the surveillance reports and the imperfect methods generally used in our hospital environment to diagnose *N. meningitidis*.

## Methods

### Study design

We conducted a retrospective, observational study (GSK study identifier : HO-17-19052) to obtain the reported number of IMD cases, hospital discharges, deaths associated with IMD and the data for serogroup characterisations in Argentina between 2007 and 2016 based on four different databases: (i) the National Clinical Surveillance System (SNVS) managed by the Health Ministry [22]; (ii) the Hospital Discharge System [23], (iii) Vital Statistics Database (VSD) under the responsibility of the National Directorate of Health Statistics [24] and (iv) the SIREVA II Laboratory network sponsored by the Pan American Health Organization (PAHO) [25, 26].

The reported data were then adjusted to account for cases potentially missed due to the partial coverage of reporting and imperfect diagnostic methods. The total numbers of IMD cases and deaths were further stratified by age groups: <1 y, 1–4 y, 5–14 y and ≥15 y.

### Burden of invasive meningococcal disease

#### Reported number of meningococcal disease

The main recorded data came from the SNVS [22]. We used this database to estimate disease burden because this is a mandatory epidemiological surveillance system that receives notifications from health care organisations of all the country using the International Statistical Classification of Diseases and Related Health Problems, 10th Revision (ICD-10) codes. The system records the number of cases of ‘meningitis and other invasive forms due to *N. meningitidis*’ (A39), ‘Haemophilus meningitis’ (G00.0), ‘Pneumococcal meningitis’ (G00.1), ‘other defined bacterial meningitis’ (G00.8) and ‘unspecified bacterial meningitis’ (G00.9) by year (2007–2016) and province, with national coverage. The total number of bacterial meningitis (G00) cases reported in this study included the number of cases under the codes G00.0, G00.1, G00.8 and G00.9.

A second database analysed was the Hospital Discharge System [23]. This is an administrative database that covers only the public hospitals of all the country, reporting discharges associated with bacterial meningitis, by age group, year (2007–2013) and province under the responsibility of the National Directorate of Health Statistics. It was originally considered to optimise our estimations. However, the analysis of this database was not deemed robust enough to include it in the main manuscript and these data are presented in Supplementary material S2.

#### Adjustments for coverage

Although reporting to the SNVS is mandatory, not all the health care organisations are compliant. As an example, the guideline for meningococcal vaccination developed by the Health Ministry reported higher numbers of IMD than the notifications included in the SNVS based only on a passive surveillance system [5, 22]. The guideline from the Health Ministry reported 289, 287, 272 and 175 IMD cases annually between 2012 and 2015 while the SNVS reported 148, 183, 175 and 116 IMD notifications for the same period. Authorities of the Health Ministry explained that the data included in the guideline for meningococcal vaccination were generated from multiple sources, including laboratory reports and outbreak notifications. Therefore, after reviewing the data from both sources of information, we observed that proportion of SNSV IMD data over total reported IMD data by the Health Ministry increased by year, being 51.2% in 2012 and 66.3% in 2015, totalling 60.8% for the whole period (2012–2015) [5]. Therefore, we estimated that overall the SNVS received notifications of only 60% of the IMD cases of the country (see Supplementary material S1 for more details) and we used that the rate (60%) for the coverage adjustment used in our analysis.

#### Adjustments for diagnostic methods

Our second adjustment aimed to mitigate the limitations related to sample processing and available methods for the quality of the IMD diagnosis currently used in Argentina. The adjustment was based on a recent three-year active surveillance study from Gentile *et al*. [20]. The data from this active surveillance study in six paediatric sentinel hospital sites across Argentina were used as a reference to calculate the ratio of IMD-confirmed cases and acute non-meningococcal bacterial meningitis (NMBM) cases identified with improved bacterial culture methods with and without the PCR for *N. meningitidis* identification. The rationale behind our adjustment is that the better the quality of the diagnostic methods used, the higher would this ratio be. All the processes involved in the diagnostic of *N. meningitidis* in this study (sample handling, transportation, time to culture seed and culture conditions) were specially considered and improved when compared to the average process in the hospitals participating in the SNVS. In addition, the inclusion of the PCR for bacterial identification increases considerably the sensitivity of the diagnostic procedure and it is missing on many hospitals reporting to the SNVS. The above-mentioned study identified 268 cases of all acute bacterial meningitis. Of them, 168 cases had positive bacterial culture results, and *N. meningitidis* was isolated in 51 cultures. Of the 100 cases with negative culture results, 30 were positive by the PCR for *N. meningitidis*. The microbiology laboratory alerts identified another 13 patients presenting unusual clinical manifestations of meningococcal disease (seven arthritis, five bacteremia and one pneumonia). In total, 94 children with meningococcal disease were confirmed [20]. Therefore, the ratio of IMD/NMBM cases in this study, using only improved bacterial culture (ratio of 0.295) or including PCR (ratio of 0.503) was calculated using equations (1) and (2), respectively:
1
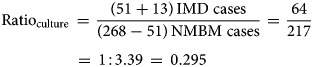

2
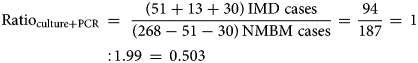


We used the above calculated ratios obtained in conditions of good quality of diagnostic methods to estimate the potential number of IMD-confirmed cases that could be identified in the SNVS, if the same procedures of the active surveillance study were used in the whole SNVS. Therefore, the data of the SNVS previously adjusted by system coverage were consecutively adjusted by diagnostic methods using the above calculated ratios. This adjustment for improved bacterial culture methods, with or without the PCR, allows for the calculation of additional *N. meningitidis* cases (i.e. IMD cases) in the SNVS every year. The additional number of IMD cases estimated for the scenario of SNVS with improved methods in the country was subtracted from the previously estimated coverage-adjusted NMBM cases to produce adjusted parameters by diagnostic methods (see Supplementary material S3 for more details). This adjustment method assumed equal reporting rates in all time periods of the analysis.

#### Incidence rates and age-distribution of IMD cases

The IRs (per 100 000 inhabitants per year) of IMD and NMBM cases were calculated by dividing the number of reported and/or estimated cases per year by the size of the population for the year considered. Population statistics were retrieved from the Comisión Económica para América Latina y el Caribe, revision 2016 [27].

To estimate the age-distribution of IMD cases for the country, the age distribution of IMD cases reported by the SIREVA II network was used over the overall adjusted number of IMD cases estimated for the SNVS in 2016.

### IMD-related deaths and case fatality ratios

Case fatality ratios (CFRs) for deaths were based on the data extracted from the VSD [24], which records the causes of death throughout Argentina using ICD-10 codes ‘meningitis and other invasive forms due to *N. meningitidis*’ (A39); ‘bacterial meningitis, not elsewhere classified’ (G00) and ‘meningitis due to other unspecified causes’ (G03), per year (2007–2015), by age group and province.

With 100% coverage of the reported IMD-related deaths to the VSD, no coverage adjustments were necessary for this database.

We used the VSD to estimate mortality rates associated with IMD (A39 code) because of the quality of the system, the recognised 100% coverage and the availability of data by age groups. The CFRs were calculated as a ratio between the number of reported IMD-related deaths as the nominator and the number of reported IMD cases by SNVS (adjusted by coverage, as previously described) as the denominator, expressed in percentages for each year.

Because of the limitation on the diagnostic methods used for *N. meningitidis* identification, the reported number of IMD-related deaths was adjusted for diagnostic methods by applying the estimated CFR to the diagnosis-adjusted number of IMD cases reported to SNVS for each year.

### Serogroup and age group stratification

The SIREVA II network (Sistema de Redes de Vigilancia de los Agentes Bacterianos Responsables de Neumonia y Meningitis) is a laboratory surveillance system implemented by the PAHO [25, 26]. We reviewed annual data (2007–2016) reported by the Argentine Reference Laboratory on the characteristics of the *N. meningitidis* isolates received from selected Argentinean hospitals. The report contains data on serogroups, age groups and sample types. Although the SIREVA II network reports data for only a limited number of IMD cases, we used this surveillance system to assess the prevalence of *N. meningitidis* serogroups in the country because of the recognised quality of the National Reference Laboratory responsible for this network and the high number of strains characterised per year.

The reported cases of IMD cases by the serogroup were analysed and reported by year as a raw number of cases (without adjustments) and percentage distribution of *N. meningitidis* serogroups. The reported data of SIREVA II in 2016 for the age groups <1 y (y); 1–4 y; 5–14 y and ≥15 y, were used to estimate the adjusted disease burden by the age-group for the country. The age-distribution of IMD cases is not reported in the bulletins of the SNVS but can be obtained from the IMD cases reported by the SIREVA II Network. We considered that the serogroup distribution reported by the NRL, appropriately represents the country scenario because of the extensive number of samples analysed per year.

## Results

### Number of notified and estimated IMD cases

Table 1 presents the number of NMBM cases (G00) and IMD cases (*N. meningitidis* infections; A39) reported to the SNVS, by year [22], and further adjusted by coverage and improved diagnostic methods. During the study period (2007–2016), the SNVS had a ratio of reported IMD/NMBM cases of 0.222, averaging 661 NMBM cases per year (IR: 1.58/100 000 per year) and 147 IMD cases per year (IR: 0.35/100 000 per year). The NMBM IRs peaked in 2008 (IR: 1.90/100 000 per year) and 2011 (1.87/100 000 per year). Reported IMD cases (A39 code) and IRs peaked in 2011 (205 cases with a rate of 0.49 per 100 000 inhabitants per year) and were the lowest in 2009 (97 cases with a rate of 0.24 per 100 000 inhabitants per year) (Table 1).

**Table 1.** Burden of NMBM and IMD cases reported to the SNVS, by year, adjusted by coverage and diagnostic methods

Number of cases per year (IRs per 100 000 inhabitants per year)	Year										
	2007	2008	2009	2010	2011	2012	2013	2014	2015	2016	Mean
Reported											
NMBM (ICD-10 code G00^a^)	703 (1.76)	765 (1.90)	531 (1.30)	644 (1.57)	778 (1.87)	699 (1.66)	662 (1.56)	682 (1.59)	617 (1.42)	526 (1.20)	**661 (1.58)**
IMD (ICD-10 code A39^b^)	142 (0.36)	155 (0.38)	97 (0.24)	137 (0.33)	205 (0.49)	148 (0.35)	183 (0.43)	176 (0.41)	116 (0.27)	111 (0.25)	**147 (0.35)**
Adjusted by coverage^c^											
Adjusted NMBM	1172 (2.94)	1275 (3.16)	885 (2.17)	1073 (2.61)	1297 (3.12)	1165 (2.77)	1103 (2.60)	1137 (2.65)	1028 (2.37)	877 (2.01)	**1101 (2.64)**
Adjusted IMD	237 (0.59)	258 (0.64)	162 (0.40)	228 (0.56)	342 (0.82)	247 (0.59)	305 (0.72)	293 (0.68)	193 (0.45)	185 (0.42)	**245 (0.59)**
Adjusted by improved bacterial culture method^d^											
Additional *Nm* identified	84 (0.21)	91 (0.23)	77 (0.19)	68 (0.17)	31 (0.08)	75 (0.18)	16 (0.04)	32 (0.08)	85 (0.20)	57 (0.13)	**62 (0.15)**
Adjusted NMBM	1088 (2.73)	1184 (2.94)	808 (1.98)	1005 (2.44)	1265 (3.04)	1090 (2.60)	1087 (2.56)	1104 (2.57)	943 (2.18)	820 (1.88)	**1040 (2.49)**
Adjusted IMD	321 (0.80)	349 (0.87)	239 (0.59)	296 (0.72)	374 (0.90)	322 (0.77)	321 (0.76)	326 (0.76)	278 (0.64)	242 (0.55)	**307 (0.74)**
Adjusted by improved bacterial culture + PCR diagnosis^d^											
Additional *Nm* identified	234 (0.59)	255 (0.63)	188 (0.46)	207 (0.50)	206 (0.50)	226 (0.54)	166 (0.39)	185 (0.43)	215 (0.50)	170 (0.39)	**205 (0.49)**
Adjusted NMBM	937 (2.35)	1020 (2.53)	696 (1.71)	866 (2.11)	1090 (2.62)	939 (2.24)	937 (2.21)	952 (2.22)	812 (1.88)	707 (1.62)	**896 (2.15)**
Adjusted IMD	472 (1.18)	513 (1.27)	351 (0.86)	435 (1.06)	549 (1.32)	473 (1.13)	471 (1.11)	478 (1.12)	409 (0.95)	355 (0.81)	**450 (1.08)**

NMBM, non-meningococcal bacterial meningitis; ICD-10, International Classification of Diseases, 10th Revision codes; IMD, invasive meningococcal disease; IR, incidence rate; Nm, Neisseria meningitidis; PCR, polymerase chain reaction; SNVS, National Clinical Surveillance System.

a ICD-10 code G00 is the addition of codes G00.0, G00.1, G00.8 and G00.9.

b ICD-10 code A39 means cases of meningitis and other invasive forms due to *N. meningitidis*.

c Adjustment by coverage considering that 60.0% of the Argentinian population is covered by the SNVS [22].

d Adjustment to account for the diagnostic methods, with a ratio of 1:3.39 (for bacterial culture diagnosis) and 1:1.99 (for bacterial culture + PCR), based on the reported data from Gentile *et al*. (2017) [20].

After coverage adjustment, assuming the notified number of cases to SNVS represented 60% of the total cases in the country, we estimated that the SNVS underreported 440 NMBM cases and 98 IMD cases in average, leading to estimated NMBM and IMD mean IRs of 2.64 and 0.59 per 100 000 inhabitants per year for NMBM and IMD, respectively (Table 1).

Improved bacterial culture methods and improved bacterial culture methods + PCR allowed us to identify an additional average number of 62 and 205 *N. meningitidis* cases (A39) per year, respectively. The estimated mean number of NMBM (G00) cases per year then decreased to an average of 1040 and 896 cases (IR: 2.49 and 2.15/100 000 per year), increasing the estimated number of IMD cases per year to 307 and 450 (IR: 0.74 and 1.08/100 000 per year) after adjusting by improved bacterial culture methods or improved bacterial culture methods + PCR, respectively (Table 1).

**Fig. 1.**
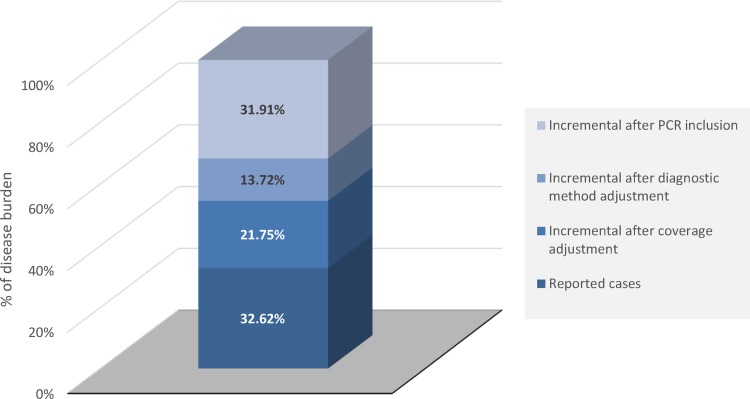
Incremental burden of IMD cases (A39) after adjustment for coverage and diagnostic methods. IMD, invasive meningococcal disease; PCR, polymerase chain reaction.

Finally, after all adjustments, the SNVS was estimated to underreport the potential number of IMD cases by approximately three times; the mean incidence for potential IMD cases per 100 000 inhabitants per year is 1.08 (Table 1). Additional increments of 21.75% of the IMD cases reported by SNVS between 2007–2016 are estimated after adjusting notified data by system coverage and additional increments of 13.72% and 31.91% after adjusting by improved bacterial culture methods and improved bacterial culture methods + PCR, respectively (Fig. 1). At the end, we calculated that the mean number of IMD cases notified in the SNVS most probably only represents 32.62% of all IMD cases in Argentina between 2007 and 2016 (Fig. 1).

### Deaths

Data of CFRs and IMD-related deaths by year, adjusted for diagnostic methods, are presented in Table 2. A mean number of 21 IMD-related deaths (mortality rate – MR: 0.051 per 100 000 inhabitants per year) were reported to VSD between 2007 and 2015, with the lowest value in 2010 (14 IMD-related deaths; MR: 0.034 per 100 000 per year) and a peak of 31 IMD-related deaths (MR: 0.076 per 100 000 per year) in 2009 (Table 2).

**Table 2.** Burden of IMD-related deaths, reported to the VSD, adjusted by diagnostic methods, by year

Number of IMD-related deaths per year (MRs; per 100 000 inhabitants per year)	Year										
	2007	2008	2009	2010	2011	2012	2013	2014	2015	Mean
Reported^a^	26 (0.065)	25 (0.062)	31 (0.076)	14 (0.034)	15 (0.036)	22 (0.052)	15 (0.035)	24 (0.056)	20 (0.046)	**21 (0.051)**
CFRs (%)^b^	11.0%	9.7%	19.2%	6.1%	4.4%	8.9%	4.9%	8.2%	10.3%	**9.2%**
Adjusted by improved bacterial culture methods^c^	35 (0.088)	34 (0.084)	46 (0.112)	18 (0.044)	16 (0.039)	29 (0.068)	16 (0.037)	27 (0.062)	29 (0.066)	**28 (0.067)**
Adjusted by improved bacterial culture + PCR diagnosis^c^	52 (0.130)	50 (0.123)	67 (0.165)	27 (0.065)	24 (0.058)	42 (0.100)	23 (0.055)	39 (0.091)	42 (0.098)	**41 (0.098)**

CFRs, case fatality ratios; IMD, invasive meningococcal disease; IRs, incidence rates; PCR, polymerase chain reaction; SNVS, National Clinical Surveillance System; VSD, Vital Statistics Database.

a Reported deaths are related to ICD-10 code A39 (meningitis and other invasive forms due to *N. meningitidis*); the system coverage for the reporting of death certificates was assumed to be 100%.

b Case fatality ratios (CFR) were calculated with the IMD-related deaths ((A39 code) reported to the VSD [24] and the number of IMD cases (A39 code) reported to the SNVS database [22], adjusted by coverage).

c To adjust the number of IMD-related (A39) deaths to account for the diagnostic methods, the number of diagnosis-adjusted IMD cases previously calculated (Table 1) was multiplied by the CFR, by year.

A mean CFR of 9.2% was calculated, with a peak of 19.2% in 2009 and the lowest being 4.4% in 2011.

After adjustment for improved bacterial culture methods and improved bacterial culture methods + PCR, we calculated that the real number of IMD-related deaths was approximately 1.30 and 1.91 times higher, respectively, than what was reported by the VSD (Table 2). The mean mortality rate of 0.051/100 000 inhabitants per year increased to 0.067 and 0.098/100 000 inhabitants per year after adjustment for bacterial culture methods and PCR, respectively (Table 2).

### Serogroup characterisation

**Fig. 2.**
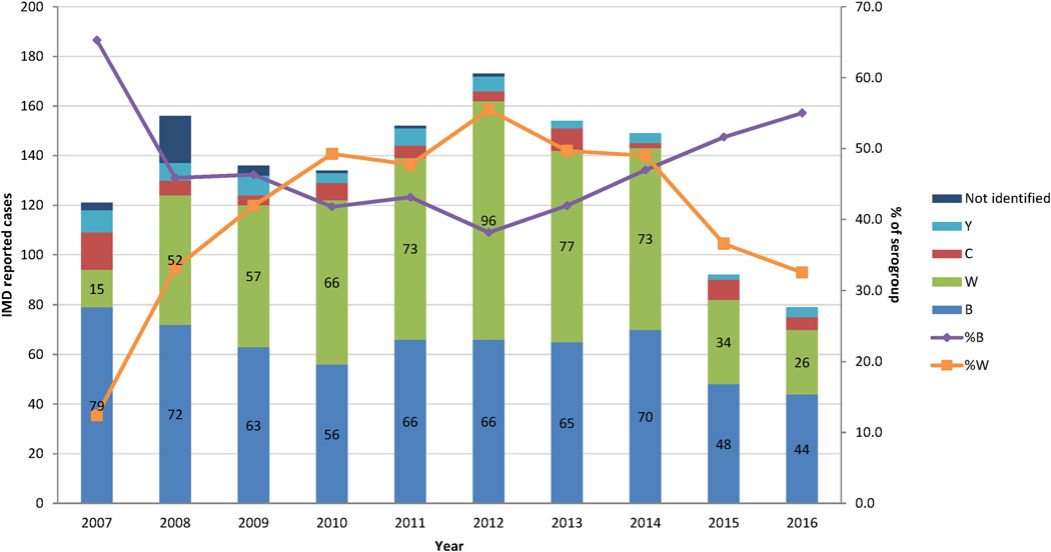
Number of IMD cases (A39) and distribution of *N. meningitidis* serogroups B and W, per year (Reported by the SIREVA II network (Sistema de Redes de Vigilancia de los Agentes Bacterianos Responsables de Neumonia y Meningitis) [25, 26]). IMD, invasive meningococcal disease.

The percentage of the two most common serogroups, B and W represented together an average of 89% of all cases of *N. meningitidis* infections characterised by the National Reference Laboratory between 2008 and 2016. Serogroup B became more common since 2012, whereas serogroup W steadily decreased over the same period (Fig. 2).

### Age stratification

**Fig. 3.**
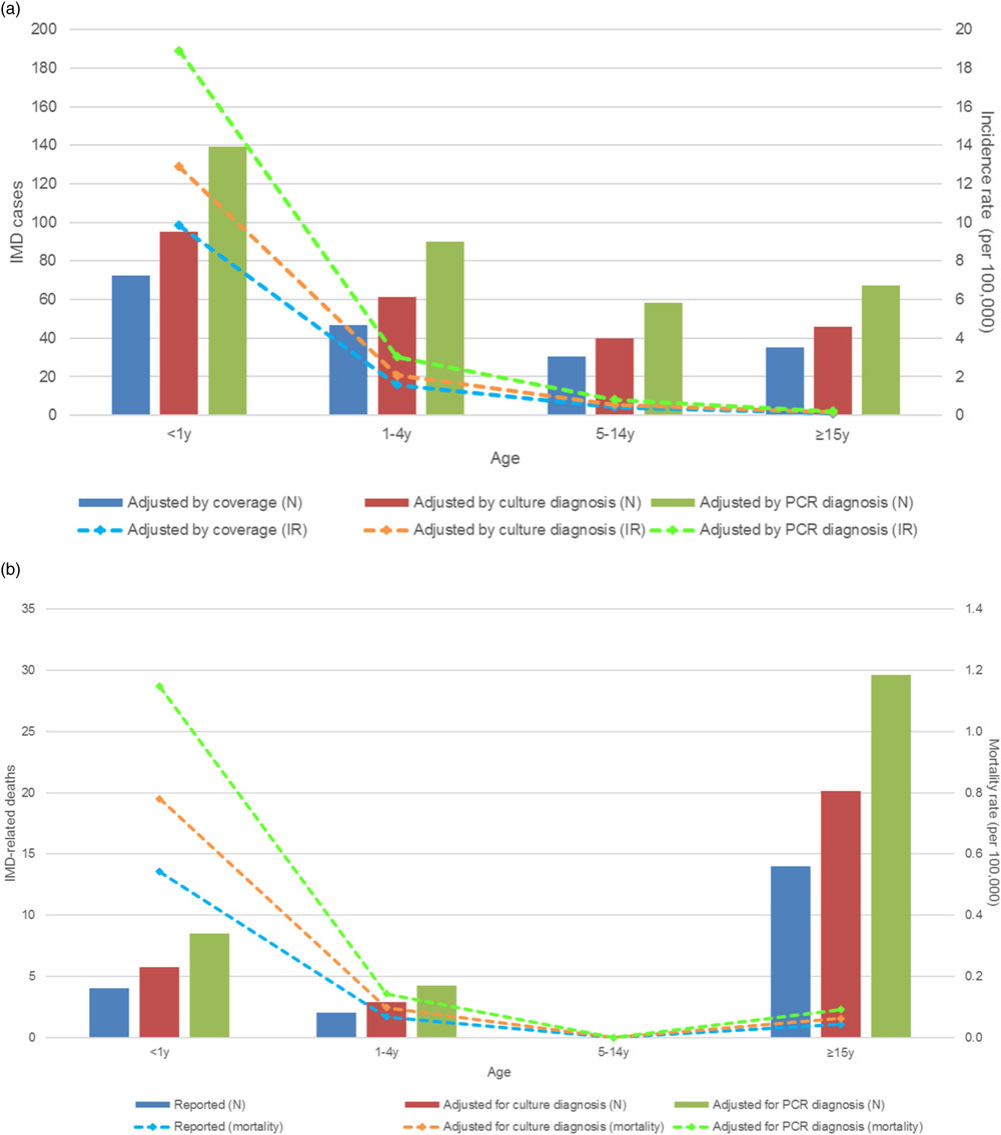
Number of cases and incidence of IMD infections in 2016 (a) and IMD-related deaths and mortality rates in 2015 (b), by age groups. IMD, invasive meningococcal disease (A39 code); IR, incidence rate; N, number of cases; PCR, polymerase chain reaction; y, year old.

The peak number and IRs of estimated IMD cases occurred in the age group <1 y with adjusted estimates of 19 cases of *N. meningitidis* per 100 000 inhabitants per year (Fig. 3a).

The number of IMD deaths was the highest in the age group ≥15 y but the mortality rate was the highest in the <1 y age group, with an estimated 1.15 IMD-related IMD deaths per 100 000 inhabitants per year. There were no deaths reported in the age group 5–14 y (Fig. 3b).

## Discussion

**Fig. 4.**
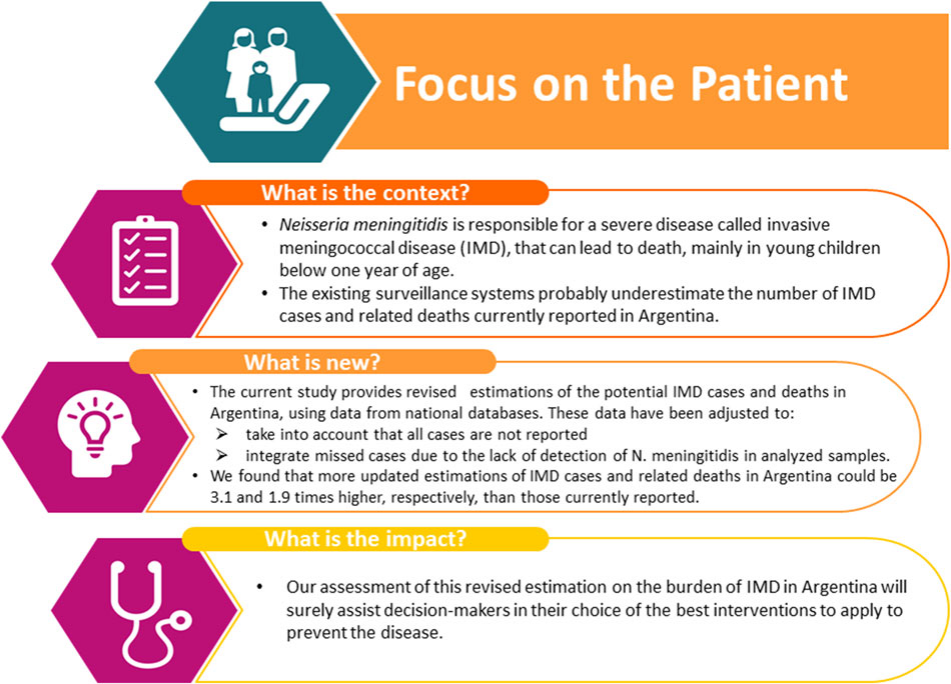
Focus on the patient.

IMD, a severe medical condition due to *N. meningitidis* bacteria, is a major public health concern but the real burden of the disease in Latin America is not exactly known and might be underreported [4]. Figure 4 presents a summary of the context, outcomes and impact of this study for health care practitioners.

Several factors can explain the limitations of the notified IMD data. First, the surveillance systems in Latin America are mainly passive, with low reporting coverage [4]. In Argentina, based on Registry of Health Institutions of the Ministry of Health (a monitoring system that records which health care organisation report case information to the SNVS) and guidelines from the Health Ministry, we estimate that only 60% of the IMD are reported to the SNVS. Second, the types of laboratory diagnostic methods available in the region are limited, which can impact the quality of sample transportation and processing; those cases are then missed from the reporting [4]. Another limitation in the IMD reporting is the diagnostic method. The previous standard for IMD case detection was the bacterial culture to identify the presence of *N. meningitidis*; however, in most cases, the rapid treatment with antibiotics of a suspected IMD case would lead to false negative cultures, impacting the correct reporting as the case would then be classified as NMBM instead of IMD case [9]. This explains why the GMI, which promotes IMD prevention worldwide, recommends the use of the PCR (and even qPCR), a diagnostic method more sensitive than classical bacterial cultures for the detection of *N. meningitidis* to confirm IMD cases, as the presence of *N. meningitidis* can still be detected after antibiotic treatment [4, 11].

Given the severity of IMD, and to improve disease prevention, it is critical to better estimate the real burden of IMD in terms of the number of cases, mortality, most affected age groups and predominant serogroups. In order to define the most realistic burden of IMD cases and fatalities in Argentina, the current study aims to remove the barriers that currently limit IMD reporting, in terms of surveillance database coverage and cases missed due to the diagnostic methods. To achieve this, we developed here an epidemiological-adjustment method to correct the effects of partial coverage of the surveillance reports and the imperfect methods used in the passive surveillance system of Argentina.

The data extracted from the database were first adjusted to account for (i) the partial coverage of the population; (ii) the impact of IMD detection by bacterial culture diagnosis and (iii) the impact of IMD detection by PCR diagnosis in addition to bacterial culture. Given the fact that IMD cases are likely reported in small outbreaks, their sporadic occurrence can be the reason for the variations observed on the incidence of IMD, mortality rates and CFRs observed among the different years analysed.

When compared to data from Europe (EU), where there is a long-standing active surveillance of IMD and definition of IMD cases is harmonised [28], our estimations of IMD burden after adjustments seem more realistic than those currently reported in Argentina. In the United Kingdom (UK), the IR of IMD has been consistently reported between 1.2 and 1.4 per 100 000 inhabitants per year for 2012–2016 [29], which is close to our estimation of 0.81 to 1.12 per 100 000 inhabitants per year for Argentina during the same period. Stratified age group data are also similar in EU where the IR of IMD is the highest in infants <1 y (8.5 per 100 000 per year for the whole EU and 11.7 per 100 000 per year in the UK, in 2016), followed by the 1–4 y age group [29]; in our study, the highest IR of IMD is estimated in the <1 y age group, with an IR of 18.90 per 100 000 inhabitants per year, followed by the 1–4 y age group. The CFR of IMD is high in EU (10%), which is comparable to our estimations for Argentina (9.2% after adjustments). None of the databases reviewed have any sign of a second peak of IMD cases in adolescents.

In Argentina, serogroups B and W were responsible for the majority of IMD. The serogroup distribution in Argentina is different from that in EU. During 2012–2016 in Argentina, the proportion of serogroup B among reported IMD cases increased and the proportion of serogroup W decreased. The opposite trends have been observed in EU during the same period where the notification rates of serogroups B and C were decreasing while W and Y serogroups were increasing. Serogroup B however remains the most represented among IMD cases in EU, followed by C and W (54%, 16% and 15%, respectively) [29].

Our study has some limitations inherent to retrospective studies. The adjustments are based on several assumptions that, even if providing from scientific evidence, could generate some uncertainty on the final results. As an example of the many limitations of this kind of study, we have used fixed factors to adjust the burden of disease for different age groups while it is obvious the surveillance system coverage is different by age groups. However, there is an alignment between our estimated data on IMD burden and those reported in other parts of the world, where active surveillance systems ensure stable and reliable data.

On the other hand, the strength of this analysis is to provide a more accurate estimation of the potential IMD burden in Argentina, based on different databases from the Health Ministry. IMD is a severe condition, particularly among young children. Our assessment of a more realistic burden of IMD in Argentina is of utmost importance for the prevention of the disease. Several IMD vaccines have been developed, each of them protecting against a different serogroup (or combination of serogroups) of *N. meningitidis*; it is crucial to set up robust and stable surveillance systems allowing the detection of changes in the disease burden and/or serogroup distribution, to assist decision-makers in the choice of prevention interventions. The purpose of this analysis is to help achieve this goal by estimating the most possible realistic burden of IMD by correcting the effects of partial coverage of the surveillance reports and the imperfect methods generally used in our hospital environment to diagnose *N. meningitidis*.

## Conclusion

In this retrospective observational study, an epidemiological-adjustment method was applied to the number of cases of IMD reported in different databases from the Health Ministry. This study helps to provide a more accurate and robust estimate of the true incidence of IMD in Argentina. After adjustments, we found that the estimated numbers of IMD cases and IMD-related deaths were 3.1 and 1.9 times higher than those currently reported to the surveillance systems, respectively.

This study provides a more realistic estimation of IMD in Argentina than the incidence currently reported, and highlights the importance of an active surveillance, with high-quality methods, for a better definition of preventive strategies against IMD in Argentina.

## Supplementary material

For supplementary material accompanying this paper visit https://doi.org/10.1017/S0950268819002024. click here to view supplementary material
