# Supplementary material for: Estimation of the real burden of invasive meningococcal disease in Argentina
Source: Epidemiol Infect. 2019 Nov 29;147:e311. doi: 10.1017/S0950268819002024 (PMC7003631; doi:10.1017/S0950268819002024)
Supplement: Supplementary file 1 [file S0950268819002024sup001.docx]

# Appendices

## Supplementary material 1. Calculation of SNVS coverage

We observed that the guideline for meningococcal vaccination developed by the Ministry of Health reports higher numbers of IMD than the notifications included in the SNVS [5,22] (Table S 1). Considering the latest four years of matching data from these two sources, the percentage of cases reported by SNVS ranged from 51% to 66%; we than considered that the SNVS received an average of 60% of the IMD cases reported in the meningococcal vaccination guideline.

### Table S1. Estimation of SNVS coverage of IMD reported cases

|  | **Number of reported IMD cases** | | | |
| --- | --- | --- | --- | --- |
|  | **2012** | **2013** | **2014** | **2015** |
| Reported by the Ministry of Health [5] | 289 | 287 | 272 | 175 |
| Reported by the SNVS [22] | 148 | 183 | 176 | 116 |
| ***Estimated SNVS coverage*** | ***51%*** | ***64%*** | ***65%*** | ***66%*** |

IMD: invasive meningococcal disease; SNVS: National Clinical Surveillance System

In addition, we also tried to adjust the data reported by the SNVS by the population covered by Health Care Organizations who report cases to the SNVS. We used the Registry of Health Institutions (RHI), [27] a monitoring system that records who is reporting case information to the SNVS. The RHI also records the type of Health Care Organization (private or public health system) and the region where they operate (province). This information allowed us to calculate the coverage linked to the reported data.

The Health Ministry reported that the private health system (support directly from individual out of pocket cost or by unions) covers 63.92% of the population and the public health system covers 36.08%.[28] The RHI reports that 1,122/1,178 (95.25%) of the institutions from the public health system and 243/1,091 (22.27%) of the institutions from the private health system (both with general and paediatric hospitalization services), report bacterial meningitis cases to the SNVS.[22] Therefore, we estimated that 48.60% of the population is covered by the Health Care Organizations reporting cases to the SNVS (leaving 51.40% of IMD cases unrepresented). Although this estimation has been confirmed by the SNVS authorities, they noted that the calculation was not adequate enough to estimate the coverage of IMD notifications because IMD is a severe disease and cases are concentrated on third-level and paediatric hospitals. This latest estimation considering the private/public sector coverage, assume an equal reporting rate in all time periods, and that all health institutions have the same risk of not reporting bacterial meningitis cases to the SNVS, which may not be fully realistic though, as there is a chance that the health facilities that do not report cases to the SNVS are the smallest health facilities and/or are located in the least affected regions. Therefore, after reviewing the data from the multiple sources of information, we decided to use a more conservative estimation of 60% coverage on IMD notifications for the SNVS, based on the calculations initially described (see Table S1).

## Supplementary material 2. Hospital Discharge System database

### Hospital Discharge System database

The Hospital Discharge System (HSD), under the National Directorate of Health Statistics, records hospital discharges associated with bacterial meningitis (BM), by age, year (2007-2013) and province and covers Argentinean public hospitals only [23]. HDS data, important when considering the burden of any disease, were initially included in the analysis to optimize our estimation of the real IMD burden in Argentina. However, coverage adjustment for hospital discharges was not deemed robust enough to include them in the main analysis; they are however presented here as supplementary material.

### Adjustments made to the data obtained from the Hospital Discharge System

As previously mentioned, only the public hospitals report data to this system. The population covered by the Public Health System by Province were used for the analysis. It was assumed that all public Health Care Organizations reported to the system; hence, only 42,0% of the BM cases were estimated to be reported to the HDS, as previously described. Therefore, we first estimated the number of hospital discharges associated to BM cases after adjustment by the coverage of the Hospital Discharge System database.

Then, we estimate the number of hospital discharges associated to IMD cases per year, by using the average ratio of IMD/NMBM (non-meningococcal bacterial meningitis) cases reported to SNVS between 2007 and 2013 (0.222; see Table 1). The estimated number of IMD cases was further adjusted to consider a scenario with good quality diagnosis methods for the country. Therefore, we adjusted the estimated number of IMD cases with a IMD/NMBM ratio of 0.295 for good bacterial culture diagnosis methods and 0.503 for the addition of PCR diagnosis, based on the reported data from Gentile et al., 2017 [20] (see details in the Methods section of the main text).

The number of NMBM (ICD-10 code G00) reported by year to the HDS between 2007 and 2013, and further adjusted by coverage and diagnostic methods (culture and PCR) are presented in Table S2.

Assuming 42.0% of BM cases were reported to the HDS database, a mean number of 1,126 BM-related hospital discharges were estimated between 2007 and 2013. The highest IMD incidence rates, adjusted for coverage, was 0.71 hospital discharges per 100,000 inhabitants in 2007 and the lowest was 0.53 hospital discharges per 100,000 in 2012 (Table S2).

Applying the 0.222 IMD/NMBM ratio, previously calculated based on the SNVS reported data between 2007-2013, we estimated a mean number of 250 IMD cases for the 2007-2013 period, after coverage adjustment (Table S2).

After considering the ratios described for good classical culture methods without and with PCR (0,295 & 0.503) for diagnosis adjustments, the number of IMD cases was calculated to be 1.26 and 1.84 times higher, respectively, than the initial IMD estimate for the HDS database with only the coverage adjustment (Table S2).

### Table S2. NMBM cases reported by the HDS database and estimated NMBM and IMD cases adjusted by coverage and diagnostic methods

| **Number of cases (IRs; per 100,000 inhabitants)** | **Year** | | | | | | | |
| --- | --- | --- | --- | --- | --- | --- | --- | --- |
|  | **2007** | **2008** | **2009** | **2010** | **2011** | **2012** | **2013** | **Mean** |
| *Reported* | | | | | | | | |
| NMBM (ICD-10 code G00) | 534 (1.34) | 515 (1.28) | 449 (1.10) | 452 (1.10) | 452 (1.09) | 424 (1.01) | 482 (1.14) | **473 (1.15)** |
| *Adjusted by coverage* | | | | | | | | |
| Adjusted NMBM*^a^* | 1,272 (3.19) | 1,227 (3.05) | 1,070 (2.63) | 1,077 (2.62) | 1,077 (2.59) | 1,010 (2.41) | 1,149 (2.71) | **1,126 (2.74)** |
| Adjusted IMD^b^ | 282 (0.71) | 272 (0.68) | 237 (0.58) | 239 (0.58) | 239 (0.57) | 224 (0.53) | 255 (0.60) | **250 (0.61)** |
| *Adjusted by culture diagnosis^c^* | | | | | | | | |
| Additional *Nm* identified | 72 (0.18) | 69 (0.17) | 61 (0.15) | 61 (0.15) | 61 (0.15) | 57 (0.14) | 65 (0.15) | **64 (0.16)** |
| Adjusted NMBM | 1,201 (3.01) | 1,158 (2.87) | 1,009 (2.48) | 1,016 (2.47) | 1,016 (2.44) | 953 (2.27) | 1,084 (2.55) | **1,062 (2.59)** |
| Adjusted IMD | 354 (0.89) | 341 (0.85) | 298 (0.73) | 300 (0.73) | 300 (0.72) | 281 (0.67) | 320 (0.75) | **313 (0.76)** |
| *Adjusted by PCR diagnosis^c^* | | | | | | | | |
| Additional *Nm* identified | 238 (0.60) | 229 (0.57) | 200 (0.49) | 201 (0.49) | 201 (0.48) | 189 (0.45) | 215 (0.51) | **211 (0.51)** |
| Adjusted NMBM | 1,035 (2.59) | 998 (2.48) | 870 (2.14) | 876 (2.13) | 876 (2.11) | 821 (1.96) | 934 (2.20) | **916 (2.23)** |
| Adjusted IMD | 520 (1.30) | 502 (1.24) | 437 (1.07) | 440 (1.07) | 440 (1.06) | 413 (0.98) | 469 (1.11) | **460 (1.12)** |

^a^ Reported NMBM cases (G00) were adjusted considering that 42.0% of the Argentinian population was covered by the HDS.

^b^ The number of IMD hospital discharges was calculated using a IMD/NMBM ratio of 0.222, corresponding to the average ratio of IMD/NMBM cases reported to the SNVS database between 2007-2013.

^c^ Adjusted according to the diagnostic methods with a ratio of 0.295 (for classical culture diagnosis) and 0.503 (for diagnosis including PCR) was used based on the reported data from Gentile et al. (2017) [20].

NMBM: non-meningococcal bacterial meningitis; HDS: Hospital Discharge System; ICD-10: International Classification of Diseases, 10^th^ Revision codes; IMD: invasive meningococcal disease; IRs: incidence rates; *Nm*: *Neisseria meningitidis*; PCR: polymerase chain reaction.

## Supplementary material 3. Adjustment performed for Good Diagnostic Methods

Our second adjustment is aimed to mitigate the limitations related to sample processing and the quality of the IMD diagnosis methods currently used in Argentina. The adjustment was based on a recent three-year active surveillance study from Gentile et al., 2017 reporting the burden of *N. meningitidis* and then producing reliable data using bacterial culture vs. culture plus PCR methods to confirm *N. meningitidis* infection and then the burden of IMD.[20] The data from this active surveillance study in 6 pediatric sentinel hospital sites across Argentina were used as a reference to calculate the ratio of IMD-confirmed cases and the acute NMBM cases identified in a study with good classic diagnosis methods (with and without PCR) for *N. meningitidis* identification. The study identified 268 probable cases of acute BM. Of them, 168 cases had positive bacterial culture results, and *N. meningitidis* was isolated in 51 cultures. Of the 100 cases with negative culture results, 30 were positive by PCR for *N. meningitidis*. The microbiology laboratory alerts identified another 13 patients presenting unusual clinical manifestations of meningococcal disease (7 arthritis, 5 bacteremia, and 1 pneumonia). In total, 94 children with meningococcal disease were confirmed. [20]

Therefore, the ratio of IMD/NMBM cases in the study, using only classical methods (ratio of 0.295) or including PCR (ratio of 0.503) was calculated using equations (1) and (2), respectively:

*(1)*${Ratio}_{culture}=\frac{\left( 51+13 \right)IMD cases}{\left( 268-51 \right)NMBM cases}=\frac{64}{217}=1:3.39=0.295$

*(2)* ${Ratio}_{culture+PCR}= \frac{\left( 51+13+30 \right)IMD cases}{\left( 268-51-30 \right)NMBM cases}=\frac{94}{187}=1:1.99=0.503$

We used these ratios to estimate the potential number of IMD-confirmed cases that could be identified in the SNVS if ideal diagnostic methods were used in the whole country. Therefore, the data of the SNVS previously adjusted by system coverage were consecutively adjusted by diagnostic methods using these ratios. This adjustment, for improved classical methods, with or without PCR, allows the detection of additional *N. meningitidis* (i.e. IMD cases). This additional number of IMD cases estimated for a scenario with improved methods in the country, was subtracted from the previously estimated coverage-adjusted NMBM cases to obtain the IMD/NMBM ratio corresponding to the diagnostic method:

$x= \frac{ratio*NM{BM}_{cov}-{IMD}_{cov}}{1+ratio}$, where:

x: number of newly *N. meningitidis* (i.e. IMD) detected via the diagnostic method.

ratio: IMD/NMBM cases ratio corresponding to the diagnostic method (0.295 for bacterial culture or 0.503 for bacterial culture with PCR)

NMBM_cov_: number of NMBM cases adjusted after surveillance system coverage

IMD_cov_: number of IMD cases adjusted after surveillance system coverage
